# Cognitive Reserve in Isolated Rapid Eye-Movement Sleep Behavior Disorder

**DOI:** 10.3390/brainsci13020176

**Published:** 2023-01-20

**Authors:** Giada D’Este, Francesca Berra, Giulia Carli, Caterina Leitner, Sara Marelli, Marco Zucconi, Francesca Casoni, Luigi Ferini-Strambi, Andrea Galbiati

**Affiliations:** 1 Department of Psychology, “Vita-Salute” San Raffaele University, 20132 Milan, Italy; 2 Sleep Disorders Center, Department of Clinical Neurosciences, Neurology, IRCCS San Raffaele Scientific Institute, 20132 Milan, Italy; 3 Department of Nuclear Medicine and Molecular Imaging, University of Groningen, University Medical Center Groningen, 9713 GZ Groningen, The Netherlands

**Keywords:** rapid-eye-movement sleep behaviour disorder, RBD, sleep disorders, cognitive reserve, Cognitive Reserve Index questionnaire, neurodegenerative disorders, α-synucleinopathies, neuropsychology, neuropsychological evaluation

## Abstract

Isolated rapid-eye-movement sleep behaviour disorder (RBD) is considered the prodromal stage of α-synucleinopathies (e.g., Parkinson’s disease and dementia with Lewy bodies); however, iRBD patients show a wide variety in the progression timing (5–15 years). The model of cognitive reserve (CR) might contribute to explaining this phenomenon. Our exploratory study aimed to evaluate, for the first time, the impact of CR level on cognitive performance in polysomnography-confirmed iRBD patients. Fifty-five iRBD patients (mean age ± SD: 66.38 ± 7.51; M/F 44/11) underwent clinical and neuropsychological evaluations at the time of diagnosis. The CR Index questionnaire was part of the clinical assessment. We found that iRBD patients with high levels of CR showed: (i) the lowest percentage of mild cognitive impairment (10%), and (ii) the best performance in visuo-constructive and verbal memory functions (i.e., the recall of the Rey–Osterrieth complex figure test). Our results suggest that CR might help iRBD patients better cope with the cognitive decline related to the neurodegenerative process, providing the first preliminary findings supporting CR as a possible protective factor in this condition. This might pave the way for future longitudinal studies to evaluate the role of CR as a modulating factor in the timing of iRBD conversion and cognitive deterioration development.

## 1. Introduction

Rapid-eye-movement (REM) sleep behaviour disorder (RBD) is a REM sleep parasomnia characterised by the loss of the physiological muscular atonia typically present during REM sleep (known as REM sleep without atonia or RSWA); as a consequence, RBD patients often act out their dreams with vocalisation and/or complex motor behaviors [1]. RBD is defined as primary or isolated (iRBD) when it occurs in the absence of any other medical conditions, or as secondary when it is caused by other neurological diseases [2]. Notably, the former is recognised worldwide as a prodromal stage of α-synucleinopathies—neurodegenerative disorders characterised by the accumulation of altered α-synuclein aggregates in the neurons and/or glial cells [3,4]. These include Parkinson’s disease (PD) and dementia with Lewy bodies (DLB), which represent the main phenoconversion endpoints of iRBD patients [5,6]. It is quite established that after 15–20 years of disease duration, the vast majority of iRBD patients (>90%) develop a full-blown α-synucleinopathy [5]. However, the time of progression is highly heterogeneous among these patients. The median time of progression varies between 12 and 14 years, but a considerable portion of iRBD patients (~33.5%) shows a faster conversion–five years of disease duration [5]. The reason underlying this inter-subject variability is still largely unknown. Both modifiable (e.g., life experience) and non-modifiable (e.g., genetic and sex) factors might modulate the speed of conversion rate in iRBD, delaying or accelerating motor and/or cognitive symptoms onset.

The cognitive reserve (CR) might play a role in this phenomenon, contributing to the delay of the phenoconversion in iRBD. Brain reserve (BR) capacity seems to protect against pathological cognitive decline, as in the case of neurodegenerative diseases [7]. Specifically, individuals with more reserve might better cope with neurodegenerative processes due to an active compensatory mechanism lying on enhanced brain network efficacy and flexibility [8]. Life-long experiences can influence the efficacy and flexibility of brain networks by promoting neural plasticity. On this basis, education, stimulating occupational experience, social environments and leisure activities can modulate the brain architecture, contributing to building up CR. In other words, individuals with a high reserve have greater neural resources, requiring more pathology burden to reach the critical threshold needed to clinically manifest ongoing pathological mechanisms (delay in the onset of clinical symptoms).

The theoretical framework of CR has been extensively investigated both in the context of normal ageing [8,9] and in neurodegenerative diseases [9,10]. Regarding PD, the literature reports that education acts as a protective factor in PD patients [11,12]. PD patients with higher education have better performances in different cognitive domains (i.e., attention, executive functions, memory and visuospatial abilities) [11]. Few studies addressed the role of CR in DLB patients, the majority focusing on education; however, results are highly heterogeneous [13,14,15,16]. One study focused on the role of occupation as a proxy of CR in DLB, showing that specific job skills (i.e., problem-solving, visual tasks and sociality) act as protective factors in DLB, shaping the connectivity of high-order neural networks [17]. Although studies on reserve are particularly significant in the preclinical stage of neurodegenerative diseases, there is no evidence regarding CR in iRBD. Recently, a cross-sectional study evaluated the possible moderating action of education in subjects diagnosed with probable RBD (questionnaire-based diagnosis) [18]. The authors demonstrated that a high level of education delays the onset of cognitive and motor decline in these patients [18].

The main aim of the present research article was to evaluate the impact of CR on the cognitive functioning of iRBD patients. Investigating the effect of long-life experience in this prodromal condition might help to explain the inter-subject variability in the progression rate. However, quantifying CR is often a hard challenge. It is increasingly clear that sustained engagement in multiple activities critically develops the CR [19]. Thus, focusing on static contributors (experience in a single period) makes it difficult to capture either the impact of prolonged exposure or the interplay of several reserve-enhancing factors [20,21]. In 2012, Nucci and colleagues [22] developed a standardised questionnaire (the Cognitive Reserve Index questionnaire (CRIq)) to measure CR in all its complexity to overcome this challenge. This tool takes into account different life experiences (education, occupation and leisure activities) and the time spent in each. Thus, for instance, it considers whether a subject changed jobs during the lifespan and weighs each experience according to the time duration. In addition, this instrument provides separate scores for each evaluated life aspect and a total score for the integration of all the variables. Thus, we used CRIq to quantify CR accumulated by individuals through their lifespan and to assess its impact on their cognitive functioning.

## 2. Materials and Methods

Fifty-five polysomnography (PSG)-confirmed iRBD patients were retrospectively recruited between 2017 and 2022 from the clinical database of the Sleep Disorders Center, San Raffaele Hospital, Milan (Italy). The clinical diagnosis of iRBD was made by a sleep-medicine expert according to the third edition of the *International Classification of Sleep Disorders* [1]. For the present study, exclusion criteria were the presence of dementia, parkinsonism or any other psychiatric or neurological condition [23,24]. All patients underwent a comprehensive clinical evaluation, including neurological examination, video-PSG exam, neuropsychological assessment, motor and non-motor symptoms questionnaires and the CRIq. All participants provided written informed consent to the experimental procedure, which was previously approved by the local ethical committee.

### 2.1. Video-PSG

Video-PSG recording included 19-channel electroencephalography placed according to the international 10–20 system [25]: electromyography of the submentalis, flexor digitorum superficialis and anterior tibialis muscles (i.e., SINBAR montage) [26]; two-channels electrooculography (i.e., right and left); electrocardiography; and airflow and respiratory-effort channels. Sleep was scored by a sleep-medicine expert according to the international guidelines of the American Academy of Sleep Medicine using 30 s epochs [27].

### 2.2. Neuropsychological Evaluation

The standard neuropsychological protocol included a comprehensive battery assessing five global cognitive domains by means of different standardised tests: (1) the cognitive screening domain was assessed by means of the mini-mental state examination (MMSE) [28]; (2) the language domain was assessed by means of the token test [29], semantic and phonemic verbal fluency tests [30] and the CAGI oral denomination test [31]; (3) the memory domain was assessed by means of the digit-span test (forward [32] and backward [33] versions), the Corsi block-tapping test [34], the Rey auditory verbal learning test (RAVLT) (i.e., immediate recall, delayed recall and recognition parts) [35] and the recall of the Rey–Osterrieth complex figure (ROCF) [36]; (4) the executive functions domain was assessed by means of Raven’s progressive matrices test (RPM) [34] and the attentive matrices test [34]; finally, (5) the visuospatial abilities domain was assessed by means of the copy of the ROCF [36]. The neuropsychological evaluation was performed by an expert neuropsychologist, who administered and scored each test according to national procedures. Specifically, raw scores obtained in each neuropsychological test were adjusted for age, gender and education, providing adjusted scores for each patient (corrected scores). Then, corrected scores were evaluated based on a five-level scale: (0) severe deficits (i.e., pathological threshold), (1) moderate deficits, (2) mild deficits, (3) minimal deficits, and (4) no deficits.

Based on this neuropsychological evaluation, the presence of mild cognitive impairment (MCI) was assessed, and a clinical diagnosis was made according to the criteria proposed by Petersen and colleagues in 2018 [37].

### 2.3. Motor and Non-Motor Symptoms Questionnaires

Motor and non-motor symptoms were evaluated through the administration of standardised questionnaires, namely the Unified Parkinson’s Disease Rating Scale (UPDRS) [38], the Non-Motor Symptoms Questionnaire (NMSQ) [39] and the Scale for Outcomes in Parkinson’s Disease—autonomic (SCOPA-AUT) [40].

### 2.4. CRIq

The CRIq was administered to each patient. It represents a standardised and reliable tool for measuring CR throughout the lifespan [22]. It investigates three different CR proxies: (i) education, i.e., years of schooling and other possible training courses; (ii) working activity, i.e., the number of years for each of the professions carried out, and the type of job scored on five degrees of intellectual demand (i.e., class 1: unskilled manual work; class 2: skilled manual work; class 3: skilled non-manual or technical work; class 4: professional occupation; class 5: highly intellectual occupation); and (iii) leisure time, including the number of years, type and frequency of different cognitively stimulating activities (i.e., intellectual, social and physical activities) [22]. The CRIq returns one composite total score and three different subscores, one for each evaluated CR proxy. Of note, the CRIq total score is classified into five different levels: level 1, low CR (less than 70); level 2, medium–low CR (70–84); level 3, medium CR (85–114); level 4, medium–high CR (115–130); and, level 5, high CR (more than 130) [22].

### 2.5. Statistical Analyses

Data were analysed using Statistical Package for Social Sciences (IBM SPSS Statistics for Windows, Version 20.0. Armonk, NY: IBM Corp.) and Jeffreys’s Amazing Statistics Program (JASP, Version 0.16.0, Amsterdam, NL) software. First, we performed the descriptive statistics to characterise the sample in terms of gender, age, level of education, presence of hyposmia, disease duration, presence of MCI, presence of motor and non-motor symptoms, and PSG indices. Corrected neuropsychological test scores were compared among patients divided according to the CRIq level. The disease duration might be a confounding variable in assessing CR modulation of neuropsychological scores; the cognitive performance of iRBD can decrease with disease progression. This requires excluding the effect of disease duration on the tested CR models. To do this, we entered disease duration as a covariate in the group comparisons. We also wanted to investigate differences across global cognitive-domain scores (rather than across single neuropsychological test scores) among subjects grouped according to their CRIq level. We first z-transformed the corrected scores of each neuropsychological test. Then, we averaged the z-scores of the neuropsychological tests belonging to a specific cognitive domain: cognitive screening, memory, language, executive functions and visuospatial abilities (see the Section 2.2 for the composition of cognitive domains). This way, we obtained global z-scores for each cognitive domain. Finally, we computed the differences in the cognitive domains among CRIq level groups, controlling for disease duration. Correlation analyses were performed between the neuropsychological tests’ corrected scores and CRIq values (total and subscores). In addition, we investigated the correlation between CRIq values and corrected neuropsychological scores with disease duration.

The Shapiro–Wilk normality test and outlier analyses were used to assess sample distribution. All the variables were normally distributed, and the statistically significant outliers were excluded from the analyses. Comparisons of parametric variables were performed using univariate ANOVA, and Bonferroni correction was applied for post-hoc pairwise comparisons. Pearson’s rho parametric test was used in all the correlation analyses. For all the analyses, *p*-values less than 0.05 were considered statistically significant.

## 3. Results

### 3.1. Clinical Characteristics of the Sample

Demographic and clinical characteristics of the whole sample (*n* = 55) are reported in Table 1. The majority of patients were males (*n* = 44; 80%) and the mean (±SD) age was 66.38 ± 7.51 years. iRBD patients showed a mean (±SD) disease duration of 4.26 ± 3.56 years. The disease duration was collected retrospectively, computing the time between subjective symptom onset and the time of diagnosis. Of note, the neuropsychological assessment was made at the time of the diagnosis. iRBD patients showed disturbed sleep (i.e., low percentage of sleep efficiency, high sleep latency and high wake after sleep onset), despite a good percentage of slow-wave sleep (mean ± SD: 20.34 ± 10.04) and REM sleep (mean ± SD: 20.38 ± 6.73). The neuropsychological evaluation demonstrated that 18 patients out of 55 were affected by MCI (32.73%), six of them had a multi-domain MCI (33.33%) and four patients presented an amnestic MCI (22.22%). iRBD patients with MCI (iRBD + MCI) were significantly (*p* = 0.049) older (mean ± SD: 69.17 ± 7.29) than iRBD patients without MCI (iRBD − MCI) (mean ± SD: 65.24 ± 7.30). With respect to motor and non-motor symptoms, patients showed a mean (±SD) UPDRS total score of 7.59 ± 5.59, a mean (±SD) SCOPA-AUT total score of 8.66 ± 6.04 and a mean (±SD) NMSS total score of 26.00 ± 21.18; moreover, approximately half of the subjects had hyposmia (*n* = 26; 50.98%).

### 3.2. Characterisation of the Sample According to CRIq

Regarding CR profiles, patients had a mean (±SD) CRIq total score of 113.33 ± 18.66; meanwhile, the mean (±SD) scores obtained in each CR proxy were as follows: education 108.04 ± 13.58, working activity 111.44 ± 17.84, and leisure time 110.65 ± 18.66 (see Table 1).

Table 2 illustrates the distribution and demographic characteristics of the patients within the different levels of the CRIq. In this context, two important points should be emphasised: (i) in the present sample, none of the patients belonged to the low CR group (level 1); and (ii) the number of subjects included in CRIq level 2 group (i.e., the lowest CRIq level in the present study) was very small since only three patients fell within this category.

### 3.3. MCI Distribution across CRIq Levels

In order to test whether higher CR levels may represent a possible protective factor against cognitive decline, the distribution of MCI within the groups was investigated. Table 3 illustrates how iRBD + MCI and iRBD − MCI patients were distributed within the four different groups of the CRIq. Interestingly, in the highest level of the CRIq most of the subjects with CRIq level 5 did not present MCI (9 out of 10, 90.00%).

### 3.4. Association between Neuropsychological Performances and CRIq Levels

Table 4 presents the results of the univariate ANOVA comparing groups defined according to CRIq levels on the mean neuropsychological scores (controlling for disease duration). Results showed significant differences in the scores obtained on the RAVLT immediate recall and the ROCF recall. However, for the RAVLT immediate recall, none of the post hoc pairwise comparisons remained significant after the Bonferroni correction. As concerns ROCF recall, post hoc comparisons showed a significant difference between CRIq level 4 group (mean ± SD: 15.41 ± 4.49) and CRIq level 5 group (mean ± SD: 21.09 ± 6.09) with a *p*-value of 0.024.

Table 5 presents the results of the univariate ANOVA comparing groups defined according to CRIq levels on the global z-scores of the five cognitive domains (controlling for disease duration). This analysis showed that the four groups differed significantly in the domains of language (*p*-value = 0.048) and memory (*p*-value = 0.032). Nevertheless, for neither the language domain nor the memory domain did any post hoc pairwise comparisons remain significant after the Bonferroni correction.

Lastly, no significant correlation was found. Indeed, correlation analyses between corrected neuropsychological scores and CRIq values (total and subscores) did not show statistically significant results. Likewise, CRIq values and corrected neuropsychological scores did not significantly correlate with disease duration.

## 4. Discussion

The present study aimed to evaluate (for the first time) the influence of CR (measured by the CRIq) on the cognitive performance of PSG-confirmed iRBD patients. iRBD represents a unique time window, ideal for testing new neuroprotective methods and better understanding α-synuclein-related neurodegenerative mechanisms. These patients have a variable timing of phenoconversion, ranging from 5 to 15 years of disease duration. Thus, evaluating the influence of CR on cognitive functioning might be helpful both to explain the inter-subject variability in the onset of cognitive symptoms and to implement strategies to postpone cognitive deterioration based on neuropsychological interventions. We found that iRBD patients with a high-level of CR showed: (i) the lowest percentage of MCI (10%), and (ii) the best performance in visuo-constructive and verbal memory (the recall of the ROCF test) functions.

The distribution of iRBD + MCI patients across CRIq levels showed a very low percentage of iRBD + MCI subjects within the CRIq level 5 group (high-level CR). This can indicate that an extremely high CR level represents a protective factor against the manifestation of cognitive decline, most likely delaying its onset. Thus, in line with the CR hypothesis [41,42], our first preliminary results suggest that CR could mitigate the impact of cognitive decline when CRIq scores are at the highest level. This finding is consistent with other studies investigating the CRIq in the context of PD and cognitive impairment, also reporting the same protective role for high CRIq scores [43,44]. In this framework, a recent study by Guzzetti and collaborators [44] investigated the role of CR in PD patients by means of the CRIq, supporting not only a protective role of CR against cognitive decline in PD, but also highlighting a prominent beneficial effect of CR at the later stages of the disease. This could provide evidence that the effect of CR in the early stages of α-synucleinopathies could not be equally strong (as demonstrated in PD) due to the time-window of reference. Moreover, it is worth noting that most iRBD patients in the present sample had a high CR level (median ± SD: 116.00 ± 18.66); consequently, there were only few subjects in the medium−low CR group (level 2) and none in the low CR group (level 1). This could have led to a ceiling effect, reducing the possibility of observing the effect of CR across all possible levels.

Comparisons of neuropsychological performances between groups classified according to CRIq levels revealed only two statistically significant differences on the RAVLT immediate recall and on the ROCF recall. Both tests belong to the memory domain, which therefore appears to be the most influenced by CRIq scores. This finding was also confirmed by comparisons between the groups on global domains scores, which showed a statistically significant difference in the memory domain, but also in the language domain (which is also implicated in the RAVLT immediate-recall performance). A link between CRIq scores and the memory domain has also emerged in several other studies focusing on other clinical populations [43,44,45,46]. This finding might be due to the fact that CRIq gives much weight to activities that promote memory rather than any other cognitive function. Specifically, the CRIq includes the education proxy, but also the working-activity proxy, which is clustered according to the degree of intellectual demand. Indeed, job classification seems to consider the academic preparation required without considering other skills that more manual jobs tend to stimulate. However, it is also important to note that almost all the comparisons did not remain statistically significant after the Bonferroni correction. For this reason, conclusions that can be drawn from these data should be taken with caution.

The present work had several limitations. First, the small sample size severely limited the interpretation of our findings. This also led to a strong imbalance in terms of numerosity for each group identified according to CRIq levels. Thus, our results need to be confirmed by future studies with larger samples equally distributed among the different CRIq levels. Second, the sample was not balanced by gender, which is partly due to the male prevalence of iRBD, but female subjects are still underrepresented. Third, the study’s cross-sectional design only provided a snapshot of a dynamic process. Given that the influence of CR on neuropsychological functioning changes with the progression of the disease [44], future longitudinal studies may provide greater insights into the timing of cognitive impairment in iRBD patients with different CR levels. Finally, the CRIq tool itself presents some issues that should be considered. Firstly, job clustering does not take into account the soft skills that some more practical jobs promote, giving almost sole weight to the academic preparation required by different jobs. Secondly, the CRIq tool was created in 2012 [22] and an update might be considered. Indeed, over the last ten years, the use of technology has occupied an increasingly important role in our lives, and therefore, its influence cannot be summarised in one generic item. For these reasons, future validation studies should consider a new, updated version of the CRIq.

To sum up, despite several methodological and practical limitations, this represents the first exploratory study investigating the role of CR in PSG-confirmed iRBD patients by means of a validated questionnaire. Conclusions that can be drawn from the present findings are limited. Still, they highlight the importance of investigating the moderating role of CR on neuropsychological functioning years before a firm diagnosis of neurodegenerative diseases. Future studies should combine CR and BR measures in order to evaluate their interactions across different stages of pathology.

## Figures and Tables

**Table 1:** Population characteristics.

	*n* = 55	%
Male	44	80.00%
Hyposmia (yes) ^a^	26	50.98%
MCI	18	32.73%
	**Mean ± SD**	**Min–Max**
Age (years)	66.38 ± 7.51	50–78
Disease duration (years)	4.26 ± 3.56	0–17
Years of education (years)	12.38 ± 4.11	5–23
Sleep latency (min)	28.18 ± 26.49	1–123
WASO (min)	70.14 ± 43.02	13–247
TST (min)	361.18 ± 58.17	221–462
%SE	78.45 ± 10.93	41.70–94.20
NAWK	16.24 ± 12.63	5–94
N1 (%)	11.66 ± 3.92	3.80–23.40
N2 (%)	48.45 ± 8.63	29.20–69.40
Slow-wave seep (%)	20.34 ± 10.04	4–51.50
REM sleep (%)	20.38 ± 6.73	6.80–39.20
REM latency (min)	99.61 ± 55.76	13.50–265
UPDRS total score ^b^	7.59 ± 5.59	0–22
SCOPA total score ^c^	8.66 ± 6.04	0–26
NMSS total score ^d^	26.00 ± 21.18	0–71
CRI—education	108.04 ± 13.58	84–140
CRI—working activity	111.44 ± 17.84	72–159
CRI—leisure time	110.65 ± 22.67	72–165
CRI—total	113.33 ± 18.66	77–156

MCI: mild cognitive impairment; SD: standard deviation; min: minutes; WASO: wake after sleep onset; TST: total sleep time; %SE: sleep efficiency; NAWK: number of awakenings; N1: stage-one sleep; N2: stage-two sleep; REM: rapid eye movement; UPDRS: Unified Parkinson’s Disease Rating Scale; SCOPA: Scales for Outcomes in Parkinson’s Disease; NMSS: Non-Motor Symptoms Scale; CRI: Cognitive Reserve Index. ᵃ Only 51 out of 55 had hyposmia information. ^b^ Only 42 out of 55 had UPDRS total score. ^c^ Only 47 out of 55 had SCOPA total score. ^d^ Only 39 out of 55 had NMSS total score.

**Table 2:** Characteristics of the groups based on CRIq levels at baseline.

CRIq Level	Cut-Off	*n* (%)	M/F	Age (Mean ± SD)	Disease Duration (Mean ± SD)
CRIq level 2	70–84	3 (5.45%)	2/1	71.25 ± 6.40	3.67 ± 1.53
CRIq level 3	85–114	25 (44.45%)	19/6	65.77 ± 7.05	3.43 ± 2.71
CRIq level 4	115–130	17 (32.73%)	14/3	64.89 ± 8.08	4.72 ± 3.85
CRIq level 5	≥130	10 (18.18%)	9/1	69.00 ± 7.18	5.27 ± 4.76

CRIq: Cognitive Reserve Index questionnaire; M: males; F: females; SD: standard deviation.

**Table 3:** MCI distribution.

CRIq Level	iRBD − MCI		iRBD + MCI	
	*n*	%	*n*	%
CRIq level 2	2	66.67%	1	33.33%
CRIq level 3	17	68.00%	8	32.00%
CRIq level 4	10	58.82%	7	41.18%
CRIq level 5	9	90.00%	1	10.00%

MCI: Mild cognitive impairment; iRBD + MCI: iRBD patients with MCI; iRBD − MCI: iRBD patients without MCI; CRIq: Cognitive Reserve Index questionnaire.

**Table 4:** Univariate ANOVA between groups identified according to CRIq level on single neuropsychological tests (controlling for disease duration).

Tests	Pathological Cut-Offs	All Patients *n* = 55	CRIq Level 2 *n* = 3	CRIq Level 3 *n =* 25	CRIq Level 4 *n =* 17	CRIq Level 5 *n =* 10	
		Mean ± SD	Mean ± SD	Mean ± SD	Mean ± SD	Mean ± SD	*p*
MMSE	<24.00	28.13 ± 1.39	27.97 ± 2.12	28.49 ± 1.41	27.91 ± 1.57	27.84 ± 0.55	0.638
Token test	<26.50	32.01 ± 1.89	33.12 ± 2.50	31.44 ± 2.30	32.51 ± 1.46	31.86 ± 1.09	0.204
Semantic fluency	<25.00	45.71 ± 8.81	49.50 ± 11.15	42.73 ± 9.92	45.61 ± 6.38	50.45 ± 7.46	0.107
Phonemic fluency	<17.00	32.13 ± 10.39	37.50 ± 16.92	29.09 ± 8.24	33.83 ± 12.68	33.45 ± 6.50	0.405
Oral naming test	<41.99	47.53 ± 0.91	47.85 ± 0.29	47.34 ± 1.14	47.39 ± 0.91	48.00 ± 0.00	ᵃ
Digit forward	<4.26	5.79 ± 1.05	6.16 ± 2.15	5.47 ± 1.09	5.89 ± 0.89	6.17 ± 0.55	0.354
Digit backward	<2.65	4.40 ± 1.08	4.56 ± 0.93	4.16 ± 0.95	4.48 ± 1.19	4.70 ± 1.20	0.642
Corsi span test	<3.46	5.21 ± 0.90	5.49 ± 0.75	5.01 ± 1.05	5.43 ± 0.88	5.12 ± 0.64	0.296
RAVLT-I	<28.53	44.08 ± 7.99	47.85 ± 9.38	40.13 ± 8.72	46.07 ± 6.04	47.34 ± 6.14	0.026 *
RAVLT-D	<4.69	9.62 ± 2.43	10.17 ± 2.35	8.87 ± 2.83	9.76 ± 2.03	10.68 ± 1.95	0.203
RAVLT-R	<8.00	13.87 ± 2.18	15.75 ± 4.35	13.36 ± 2.63	14.00 ± 0.84	14.00 ± 1.48	0.221
ROCF-C	<28.88	32.29 ± 3.28	32.31 ± 6.72	32.26 ± 3.05	31.72 ± 3.30	33.25 ± 2.22	0.648
ROCF-R	<9.47	17.22 ± 5.37	20.37 ± 5.45	16.19 ± 4.73	15.41 ± 4.49	21.09 ± 6.09	0.029 *
RPM	<18.00	29.70 ± 4.20	33.87 ± 2.87	28.75 ± 4.58	29.53 ± 3.92	30.39 ± 3.60	0.066
Attentive matrices	<31.00	49.06 ± 5.24	52.00 ± 5.50	49.62 ± 4.24	47.75 ± 6.35	49.02 ± 5.07	0.264

CRIq: Cognitive Reserve Index questionnaire; SD: standard deviation; MMSE: mini-mental state Examination; RAVLT-I: Rey auditory verbal learning test-immediate; RAVLT-D: Rey auditory verbal learning test—delayed; RAVLT-R: Rey auditory verbal learning test—recognition; ROCF-C: Rey–Osterrieth complex figure—copy; ROCF-R: Rey–Osterrieth complex figure—recall; RPM: Raven’s progressive matrices. ᵃ The variance in oral naming test is equal to 0 after grouping by CRIq level. * *p*-value < 0.05.

**Table 5:** Univariate ANOVA between groups identified according to CRIq level on global neuropsychological performances (controlling for disease duration).

Cognitive Domain	CRIq Level 2	CRIq Level 3	CRIq Level 4	CRIq Level 5	
	Mean ± SD	Mean ± SD	Mean ± SD	Mean ± SD	*p*
Cognitive screening	−0.11 ± 1.51	0.26 ± 0.99	0.15 ± 1.12	−0.20 ± 0.39	0.538
Language	0.40 ± 0.69	−0.22 ± 0.63	0.08 ± 0.42	0.25 ± 0.32	0.048 *
Memory	0.36 ± 0.65	−0.19 ± 0.58	0.06 ± 0.31	0.25 ± 0.38	0.032 *
Executive functions	0.67 ± 0.96	−0.02 ± 0.66	−0.05 ± 0.54	0.05 ± 0.37	0.122
Visual spatial abilities	0.29 ± 2.02	0.28 ± 0.92	0.12 ± 0.99	0.57 ± 0.67	0.458

CRIq: Cognitive Reserve Index questionnaire; SD: standard deviation; * *p*-value < 0.05.

## Data Availability

The conditions of our ethics approval do not permit public archiving of anonymised study data. Readers seeking access to the data should contact the corresponding author. Access will be granted to named individuals in accordance with ethical procedures governing the reuse of sensitive data. Specifically, requestors must complete a formal data-sharing agreement.

## References

[1] American Academy of Sleep Medicine (2014). International Classification of Sleep Disorders.

[2] Högl B., Stefani A., Videnovic A. (2018). Idiopathic REM sleep behaviour disorder and neurodegeneration—An update. Nat. Rev. Neurol..

[3] Spillantini M.G., Schmidt M.L., Lee V.M.-Y., Trojanowski J.Q., Jakes R., Goedert M. (1997). α-Synuclein in Lewy bodies. Nature.

[4] Spillantini M.G., Crowther R.A., Jakes R., Hasegawa M., Goedert M. (1998). α-Synuclein in filamentous inclusions of Lewy bodies from Parkinson’s disease and dementia with Lewy bodies. Proc. Natl. Acad. Sci. USA.

[5] Galbiati A., Verga L., Giora E., Zucconi M., Ferini-Strambi L. (2019). The risk of neurodegeneration in REM sleep behavior disorder: A systematic review and meta-analysis of longitudinal studies. Sleep Med. Rev..

[6] Postuma R.B., Iranzo A., Hu M., Högl B., Boeve B.F., Manni R., Oertel W.H., Arnulf I., Ferini-Strambi L., Puligheddu M. (2019). Risk and predictors of dementia and parkinsonism in idiopathic REM sleep behaviour disorder: A multicentre study. Brain.

[7] Barulli D., Stern Y. (2013). Efficiency, capacity, compensation, maintenance, plasticity: Emerging concepts in cognitive reserve. Trends Cogn. Sci..

[8] M. Tucker A., Stern Y. (2011). Cognitive reserve in aging. Curr. Alzheimer Res..

[9] Stern Y. (2012). Cognitive reserve in ageing and Alzheimer’s disease. Lancet Neurol..

[10] Xu W., Yu J.-T., Tan M.-S., Tan L. (2015). Cognitive reserve and Alzheimer’s disease. Mol. Neurobiol..

[11] Hindle J.V., Martyr A., Clare L. (2014). Cognitive reserve in Parkinson’s disease: A systematic review and meta-analysis. Park. Relat. Disord..

[12] Cubo E., Rojo A., Ramos S., Quintana S., Gonzalez M., Kompoliti K., Aguilar M. (2002). The importance of educational and psychological factors in Parkinson’s disease quality of life. Eur. J. Neurol..

[13] Perneczky R., Häussermann P., Drzezga A., Boecker H., Granert O., Feurer R., Förstl H., Kurz A. (2009). Fluoro-deoxy-glucose positron emission tomography correlates of impaired activities of daily living in dementia with Lewy bodies: Implications for cognitive reserve. Am. J. Geriatr. Psychiatry.

[14] Perneczky R., Drzezga A., Boecker H., Ceballos-Baumann A.O., Granert O., Förstl H., Kurz A., Häussermann P. (2008). Activities of daily living, cerebral glucose metabolism, and cognitive reserve in Lewy body and Parkinson’s disease. Dement. Geriatr. Cogn. Disord..

[15] Perneczky R., Häussermann P., Diehl-Schmid J., Boecker H., Förstl H., Drzezga A., Kurz A. (2007). Metabolic correlates of brain reserve in dementia with Lewy bodies: An FDG PET study. Dement. Geriatr. Cogn. Disord..

[16] Lamotte G., Morello R., Lebasnier A., Agostini D., Bouvard G., De La Sayette V., Defer G.L. (2016). Influence of education on cognitive performance and dopamine transporter binding in dementia with Lewy bodies. Clin. Neurol. Neurosurg..

[17] Carli G., Boccalini C., Vanoli G., Filippi M., Iannaccone S., Magnani G., Perani D. (2021). Specific occupational profiles as proxies of cognitive reserve induce neuroprotection in dementia with Lewy bodies. Brain Imaging Behav..

[18] Chen M., Chen J., Xu X., Qiao F., Wang X., Ji S., Gu Z., Chhetri J.K., Chan P. (2020). Education Moderates the Association of Probable REM Sleep Behavior Disorder With Cognitive and Motor Impairments in Community-Dwelling Older People. Front. Neurol..

[19] Richards M., Deary I.J. (2005). A life course approach to cognitive reserve: A model for cognitive aging and development?. Ann. Neurol. Off. J. Am. Neurol. Assoc. Child Neurol. Soc..

[20] Fratiglioni L., Paillard-Borg S., Winblad B. (2004). An active and socially integrated lifestyle in late life might protect against dementia. Lancet Neurol..

[21] Reed B.R., Dowling M., Farias S.T., Sonnen J., Strauss M., Schneider J.A., Bennett D.A., Mungas D. (2011). Cognitive activities during adulthood are more important than education in building reserve. J. Int. Neuropsychol. Soc..

[22] Nucci M., Mapelli D., Mondini S. (2012). Cognitive Reserve Index questionnaire (CRIq): A new instrument for measuring cognitive reserve. Aging Clin. Exp. Res..

[23] Postuma R.B., Berg D., Stern M., Poewe W., Olanow C.W., Oertel W., Obeso J., Marek K., Litvan I., Lang A.E. (2015). MDS clinical diagnostic criteria for Parkinson’s disease. Mov. Disord..

[24] American Psychiatric Association (2013). Diagnostic and Statistical Manual of Mental Disorders.

[25] Klem G.H. (1999). The ten-twenty electrode system of the international federation. the internanional federation of clinical nenrophysiology. Electroencephalogr. Clin. Neurophysiol. Suppl..

[26] Frauscher B., Iranzo A., Gaig C., Gschliesser V., Guaita M., Raffelseder V., Ehrmann L., Sola N., Salamero M., Tolosa E. (2012). Normative EMG values during REM sleep for the diagnosis of REM sleep behavior disorder. Sleep.

[27] Berry R.B., Brooks R., Gamaldo C.E., Harding S.M., Marcus C.L., Vaughn B.V. (2020). The AASM Manual for the Scoring of Sleep and Associated Events, Version 2.6.

[28] Measso G., Cavarzeran F., Zappalà G., Lebowitz B.D., Crook T.H., Pirozzolo F.J., Amaducci L.A., Massari D., Grigoletto F. (1993). The mini-mental state examination: Normative study of an Italian random sample. Dev. Neuropsychol..

[29] De Renzi E., Faglioni P. (1978). Normative data and screening power of a shortened version of the Token Test. Cortex.

[30] Novelli G., Papagno C., Capitani E., Laiacona M. (1986). Tre test clinici di ricerca e produzione lessicale. Taratura su sogetti normali. Arch. Psicol. Neurol. Psichiatr..

[31] Catricala E., Della Rosa P.A., Ginex V., Mussetti Z., Plebani V., Cappa S.F. (2013). An Italian battery for the assessment of semantic memory disorders. Neurol. Sci..

[32] Orsini A., Grossi D., Capitani E., Laiacona M., Papagno C., Vallar G. (1987). Verbal and spatial immediate memory span: Normative data from 1355 adults and 1112 children. Ital. J. Neurol. Sci..

[33] Kessels R.P.C., van Den Berg E., Ruis C., Brands A.M.A. (2008). The backward span of the Corsi Block-Tapping Task and its association with the WAIS-III Digit Span. Assessment.

[34] Spinnler H., Tognoni G. (1987). Italian Group on the Neuropsychological Study of Ageing: Italian standardization and classification of neuropsychological tests. Ital. J. Neurol. Sci..

[35] Carlesimo G.A., Caltagirone C., Gainotti G., Fadda L., Gallassi R., Lorusso S., Marfia G., Marra C., Nocentini U., Parnetti L. (1996). The mental deterioration battery: Normative data, diagnostic reliability and qualitative analyses of cognitive impairment. Eur. Neurol..

[36] Caffarra P., Vezzadini G., Dieci F., Zonato F., Venneri A. (2002). Rey-Osterrieth complex figure: Normative values in an Italian population sample. Neurol. Sci..

[37] Petersen R.C., Lopez O., Armstrong M.J., Getchius T.S.D., Ganguli M., Gloss D., Gronseth G.S., Marson D., Pringsheim T., Day G.S. (2018). Practice guideline update summary: Mild cognitive impairment: Report of the Guideline Development, Dissemination, and Implementation Subcommittee of the American Academy of Neurology. Neurology..

[38] Goetz C.G., Tilley B.C., Shaftman S.R., Stebbins G.T., Fahn S., Martinez-Martin P., Poewe W., Sampaio C., Stern M.B., Dodel R. (2008). Movement Disorder Society-sponsored revision of the Unified Parkinson’s Disease Rating Scale (MDS-UPDRS): Scale presentation and clinimetric testing results. Mov. Disord. Off. J. Mov. Disord. Soc..

[39] Chaudhuri K.R., Martinez-Martin P., Schapira A.H.V., Stocchi F., Sethi K., Odin P., Brown R.G., Koller W., Barone P., MacPhee G. (2006). International multicenter pilot study of the first comprehensive self-completed nonmotor symptoms questionnaire for Parkinson’s disease: The NMSQuest study. Mov. Disord. Off. J. Mov. Disord. Soc..

[40] Visser M., Marinus J., Stiggelbout A.M., Van Hilten J.J. (2004). Assessment of autonomic dysfunction in Parkinson’s disease: The SCOPA-AUT. Mov. Disord. Off. J. Mov. Disord. Soc..

[41] Stern Y. (2009). Cognitive reserve. Neuropsychologia.

[42] Perneczky R., Kempermann G., Korczyn A.D., Matthews F.E., Ikram M.A., Scarmeas N., Chetelat G., Stern Y., Ewers M. (2019). Translational research on reserve against neurodegenerative disease: Consensus report of the International Conference on Cognitive Reserve in the Dementias and the Alzheimer’s Association Reserve, Resilience and Protective Factors Professional Interest Area working groups. BMC Med..

[43] Devos H., Gustafson K.M., Liao K., Ahmadnezhad P., Kuhlmann E., Estes B., Martin L., Mahnken J., Brooks W., Burns J. (2022). Effect of cognitive reserve on physiological measures of cognitive workload in older adults with cognitive impairments. medRxiv.

[44] Guzzetti S., Mancini F., Caporali A., Manfredi L., Daini R. (2019). The association of cognitive reserve with motor and cognitive functions for different stages of Parkinson’s disease. Exp. Gerontol..

[45] Krch D., Frank L.E., Chiaravalloti N.D., Vakil E., DeLuca J. (2019). Cognitive reserve protects against memory decrements associated with neuropathology in traumatic brain injury. J. Head Trauma Rehabil..

[46] Sandry J., DeLuca J., Chiaravalloti N. (2015). Working memory capacity links cognitive reserve with long-term memory in moderate to severe TBI: A translational approach. J. Neurol..

